# Pharmacist management of atrial fibrillation in UK primary care: a cross-sectional study

**DOI:** 10.1080/20523211.2024.2321592

**Published:** 2024-03-20

**Authors:** Shahd Al-Arkee, Julie Mason, M. Sayeed Haque, Abdullah Alshehri, Zahraa Jalal

**Affiliations:** a Institute of Clinical Sciences, University of Birmingham, Birmingham, United Kingdom; b Institute of Applied Health Research, University of Birmingham, Birmingham, United Kingdom; c Department of Clinical Pharmacy, Taif University, Taif, Saudi Arabia

**Keywords:** Pharmacist, management, atrial fibrillation, primary care, general practice, community pharmacy, quantitative study

## Abstract

**Background:**

Atrial Fibrillation (AF) increases the risk of stroke by a factor of five, leading a significant cost burdens on healthcare system. Pharmacists, especially those based in a primary care environment are well placed to support patients in this therapeutic area.

**Objectives:**

To assess primary care pharmacists’ actual knowledge on the management of AF symptoms and anticoagulation. Furthermore, to investigate the resources used by pharmacists.

**Methods:**

A cross-sectional study using survey was conducted, targeting UK-based registered pharmacists employed within primary care settings. Quantitative data were analysed utilising descriptive univariate and bivariate statistics.

**Results:**

349 pharmacists completed the adapted 19-questions of the pharmacists’ knowledge. Out of a maximum of 19 points, the mean score was 14.34 ± 2.2 (75 ± 11.6%). The questionnaire revealed several significant gaps in pharmacists’ knowledge. Most of the surveyed pharmacists (62.8%) reported that they used sources of information to support their consultations. Half reported that they used the National Institute for Health and Care Excellence (NICE) guidance (52.4%) and the British National Formulary (BNF) (50.7%).

**Conclusions:**

Primary care pharmacists are knowledgeable about AF and its management; however, some gaps exist which may require addressing. Although pharmacists use a variety of information resources, it is the traditional resources that remain the most frequently used.

## Background

Atrial Fibrillation (AF) is the most commonly diagnosed cardiac arrhythmia. In the UK, approximately 2% of the population has been diagnosed with AF and a sustained increase in prevalence is predicted in the future (National Institute for Health and Care Excellence [NICE], 2021). AF is associated with a 5-fold increased risk of stroke (Wolf et al., 1987), leading a significant cost burdens on healthcare system. The direct costs of AF in the UK account for approximately 1% of overall healthcare spending, driven by AF-related complications (e.g. stroke) and treatment costs (e.g. hospitalisations) (Stewart et al., 2004). The costs will rise in line with prevalence unless AF is prevented and treated (Kirchhof et al., 2016).

For the prevention of AF-related stroke, oral anticoagulants (OACs), such as vitamin K antagonists (VKAs) and direct oral anticoagulants (DOACs) have been shown as effective agents. VKAs, mainly warfarin, have been shown to reduce the risk of stroke by 60% in patients with AF (Hart et al., 2007) and DOACs have been proven as non-inferior in the stroke prevention when compared to warfarin (Connolly et al., 2009; Giugliano et al., 2013; Granger et al., 2011; Patel et al., 2011). Healthcare professionals (HCPs) including pharmacists are important stakeholders to ensure consistent anticoagulation management (Kirchhof et al., 2016). For pharmacist warfarin management, studies report to have a positive impact on outcomes such as increases in amount of time International Normalised Ratio (INR) values are within therapeutic range (Gupta et al., 2015), increased patient understanding of their medication (Collins et al., 2014), and reduced anticoagulation-related emergency department visits (Rudd & Dier, 2010). Further, studies report pharmacists DOACs management to have impact on increasing appropriate dosing of DOAC (Ashjian et al., 2017), and medication adherence (Shore et al., 2015). Thus far, evidence suggests that pharmacists may have a significant role in anticoagulation management. However, a multinational survey indicated that pharmacists did not always feel confident in supporting patients on anticoagulation therapy (Papastergiou et al., 2017). Another survey reported that pharmacists were more confident in their knowledge (i.e. approximately 40% of pharmacists felt uncertain on NOAC comparable to 23% for VKA), and access to resources for VKAs than for DOACs (Hamedi et al., 2017). Similarly, a recent survey showed pharmacists more confident in providing pharmaceutical care on warfarin (*n* = 479, 88.3%) compared DOACs (*n* = 211, 38.9%) (*P *< .001) (Tan et al., 2021). These quantitative studies explored pharmacists’ knowledge, focussed on the self-reported confidence in knowledge in this therapeutic area. However, there is a paucity of published evidence regarding the actual knowledge of pharmacists on AF and anticoagulation management, and the resources used to support their clinical role in practice. This study assesses community and GP pharmacists’ actual knowledge on how to advise on the management of AF symptoms and anticoagulation. Furthermore, this study investigates the resources used by pharmacists to support anticoagulation management in patients with AF within daily practice.

## Methods

### Study design

The study design was a national cross-sectional study using survey of primary care pharmacists working in community pharmacies and/or general practices (GPs) in the UK.

### Questionnaire development

This study used a questionnaire comprised of two sections (Additional file 1).

*Section one:* This section was developed to better understand certain background characteristics of pharmacists, and included demographic data including gender, years of experience, professional sector, and if participants had undertaken any independent prescribing courses or postgraduate courses. It was designed with a list of options provided. The section was also developed to achieve the secondary aim of this study, i.e. the identification of resources used by pharmacists to support the management of AF. Questions were designed as multiple-choice; a list of resources was provided and included an option of ‘other’ to allow open ended responses.

*Section two*: This was designed to achieve the primary aim of this study which was the assessment of pharmacists’ knowledge. The section consisted of 19-questions, which included 8 questions about AF in general; 5 about OACs; 3 about VKAs; and 3 about DOACs. All questions were designed as multiple-choice questions, having only one correct answer. An option to choose ‘I do not know’ was also included to avoid pharmacists guessing answers. The pharmacists’ knowledge questionnaire was developed based on (1) A validated questionnaire used for knowledge assessment of AF management and anticoagulation in patients with AF (Desteghe et al., 2016); and (2) Clinical recommendations of the national guidance (National Institute for Health and Care Excellence [NICE]) on the diagnosis and management of AF (NICE, 2021). The original questionnaire (Desteghe et al., 2016) was adapted to be eligible to use in this research, as it was originally validated with different populations (i.e. patients with AF, other than with pharmacists) and in countries outside the UK (i.e. European countries), and for this reason, it was crucial to adapt it according to the UK guidelines.

### Sample size

Sample size estimation was based on existing literature with similar methodology (i.e. suitable research approach to address the research problem) and nature (i.e. similar targeted populations) (Hamedi et al., 2017).

### Ethics approval

This study was given a favourable opinion on 5 September 2019 by the University of Birmingham (UOB) Research Ethics Committee (Reference ERN_19-0908). Informed consent was obtained from every participant. Before signing the consent form, participants received an information participant sheet that described the nature and purpose of the study. Participants’ data was protected according to the General Data Protection Regulation (Information Commissioner’s Office, 2018), and confidentiality and anonymity were maintained for this research process.

### Piloting questionnaire

Following ethical approval, the piloting study was initiated. A two-stage process was used to pilot the questionnaire. First, the paper version of the questionnaire was piloted by 10 pharmacists to examine its clarity, applicability, and comprehensibility. The main researcher (SA) visited different pharmacies located in the West Midlands in order to complete the first stage. This feedback resulted in a minor amendment to incorporate the option ‘other’ to one question. For the second stage of piloting, an online version of the questionnaire was created using JISC Online Surveys, licensed to the University of Birmingham, and further piloted by 10 other pharmacists to examine online usability and functionality. The online version was chosen as the circulation of the survey was more convenient. The results from the piloting stage included in the final data analysis as there was no fundamental amendment to the questionnaire and the data was highly relevant to the research question. This method was supported by Peat et al. as a basic concept for quantitative methods in health science research (Peat et al., 2020).

### Data collection

The survey was allowed to run from December 2019 until March 2020 for piloting the questionnaire; and from August 2020 until September 2021 for the data collection. The survey targeted UK-based registered pharmacists employed within primary care settings i.e. community pharmacies and general practices (GPs). Both paper and online formats were used, with the paper version distributed during pharmacy visits, and the online version circulated using various social media platforms (LinkedIn, Twitter and Telegram). The questionnaire suitable for online platforms using both smartphones and computers.

### Statistical analysis

Data analysis comprised descriptive univariate and bivariate statistics. Continuous data was reported as means (standard deviation) or median (interquartile range), as appropriate. Categorical data was reported as frequencies and percentages. Normal distribution of variables was determined using the Shapiro–Wilk W test and Kolmogorov–Smirnov test. Comparison between pharmacists’ groups was performed using the Mann–Whitney test and Spearman’s Rho test. The data was separated by ‘prescriber status’ to enable a balance in participant numbers (i.e. in a way that the non-independent prescribers group included both non-independent prescribers and supplementary prescribers). The data was also separated by the ‘professional sector’ (i.e. community and GP) to facilitate the assessing of pharmacists’ knowledge in each sector, without including pharmacists who work in cross-sector (i.e. community and academia or GP and academia). Incorporating academic pharmacists into this particular analysis could be a bias, as they generally may have a high level of knowledge.

Responses to the knowledge section were dichotomised, with correct answers scored as 1 point and incorrect or ‘I do not know’ answers scored as zero. The total score on this section was calculated out of a maximum of 19 points. Statistical significance of the difference in knowledge score was assessed at *P-value *< .05. Statistical analysis was performed using Statistical Package for the Social Sciences (SPSS) 27.0 (IBM Corp., Armonk, N.Y., USA). An overall summary was generated using GraphPad-Prism 9.0.0 (GraphPad software, San Diego, California, USA) (GraphPad, 2022). The analysis was performed by the main researcher (SA), and the UOB statistician (SH) reviewed the analysis process; and the interpretation was verified by other researchers (JM, AA and ZJ).

## Results

### Response rate

The use of social media to actively circulate the questionnaire made identifying the sample size of the targeted population (i.e. pharmacists who viewed the survey) problematic, and hence it was not possible to calculate the response rate. For the paper copy version, 23 valid responses were received out of 24 responses requested. In total, there were 349 responses to the survey.

### Demographic data

The majority of participants were female 67.9% (237). Participants’ length of experience ranged from 0.5–44 years (mean 15.7, median 15.0 years). Professional cross-sector experience varied widely including academia, community, GP, hospital, and industry. The proportion of 43.6% (152) of all participants were independent prescribers, with 18.1% (63) specialised in CVD. Since registering as UK pharmacists, none of the pharmacists had undertaken specific training on AF and associated anticoagulation management. The proportion of 38.1% (133) reported that they had only undertaken general training to develop their consultation skills. Further participant demographics can be seen in (Table 1).

**Table 1.** Demographic characteristics of the primary care pharmacists (*n* = 349).

Characteristics	Participants % (*n*)
**Gender**	
Male	30.9 (108)
Female	67.9 (237)
Other	0.3 (1)
Prefer not to say	0.9 (3)
**Professional experience (Cross-sector)**	
Academia	4.9 (17)
Community	51.0 (178)
General practice	55.6 (194)
Hospital	4.3 (15)
Industry	1.7 (6)
**Prescriber status**	
Independent prescriber (IP)^a^	43.6 (152)
**Specialist area**	
Blood and nutrition	0.3 (1)
Cardiovascular system	18.1 (63)
Ear, nose and oropharynx	0.3 (1)
Endocrine system	4.3 (15)
Immune system and malignant disease	0.6 (2)
Infection	1.4 (5)
Musculoskeletal system	1.5 (5)
Nervous system	0.9 (3)
Respiratory system	6.3 (22)
Other	10.0 (35)
**Qualifications post IP registration**	
Diploma	30.1 (105)
Masters	13.2 (46)
Doctorate	2.0 (7)
Non-independent prescriber	56.4 (197)
Supplementary prescriber	1.1 (4)
Non-prescriber *	55.3 (193)
**Post-graduate (PG)**^b^ **qualifications**	
Diploma	12.9 (45)
Masters	4.5 (17)
Doctorate	1.1 (4)
**Participation in PG**^b^**consultation skills courses**	38.1 (133)

^a^ IP: Independent prescriber; ^b^PG: Post-graduate.

*Non-prescriber: Those that did not have a prescribing qualification.

### Knowledge of AF and its anticoagulation management

**Figure 1.**
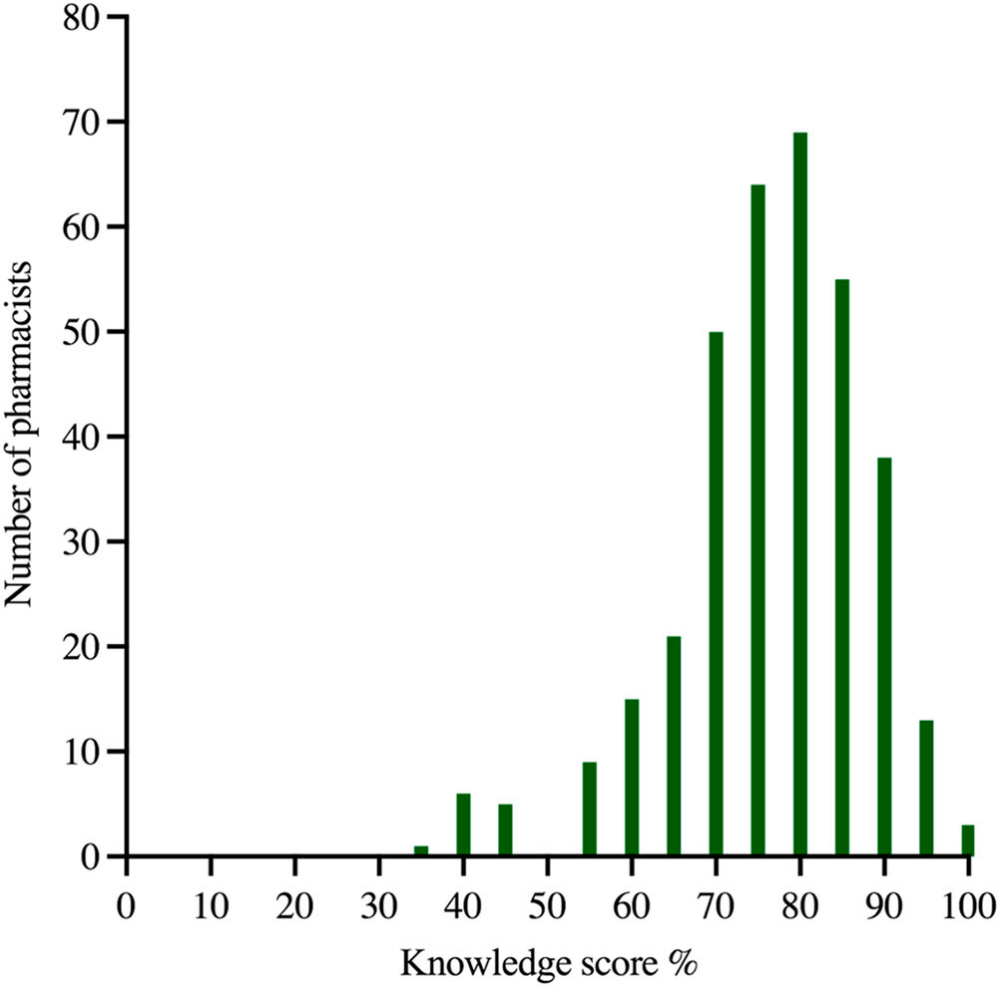
Frequency distribution of scores (%) for pharmacists’ knowledge (*n* = 349).

A total of 349 pharmacists completed the 19-questions of the pharmacists’ knowledge. Out of a maximum of 19 points, the mean score for the pharmacists’ knowledge was 14.34 ± 2.2 (75 ± 11.6%), with a minimum score of 7 (36.8%) and a maximum score of 19 (100%) (Figure 1).

**Figure 2.**
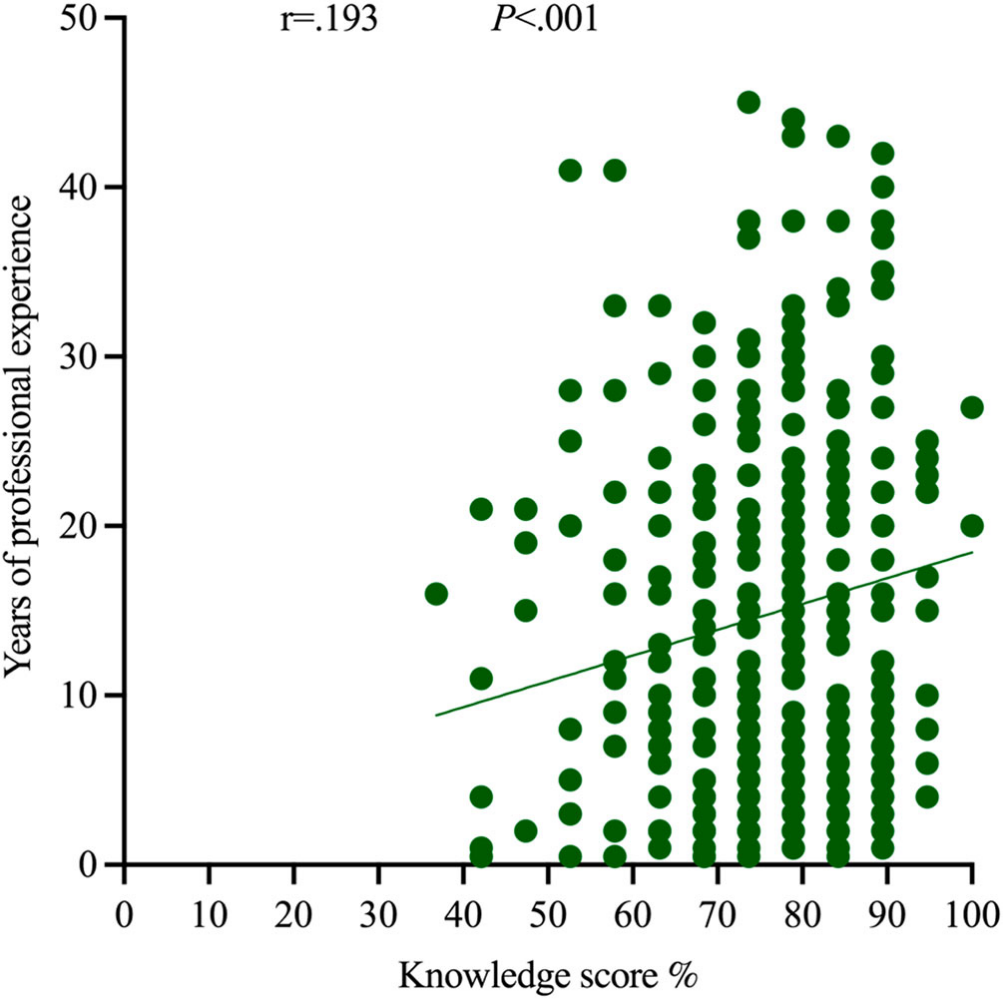
Correlation coefficient of scores (%) for pharmacists’ knowledge (*n* = 349).

The score on the pharmacists’ knowledge correlated with years of professional experience, and a direct relation was reported (i.e. positively and fairly weak relation) (r = .193, *P *< .001, *n* = 349) (Figure 2).

**Figure 3.**
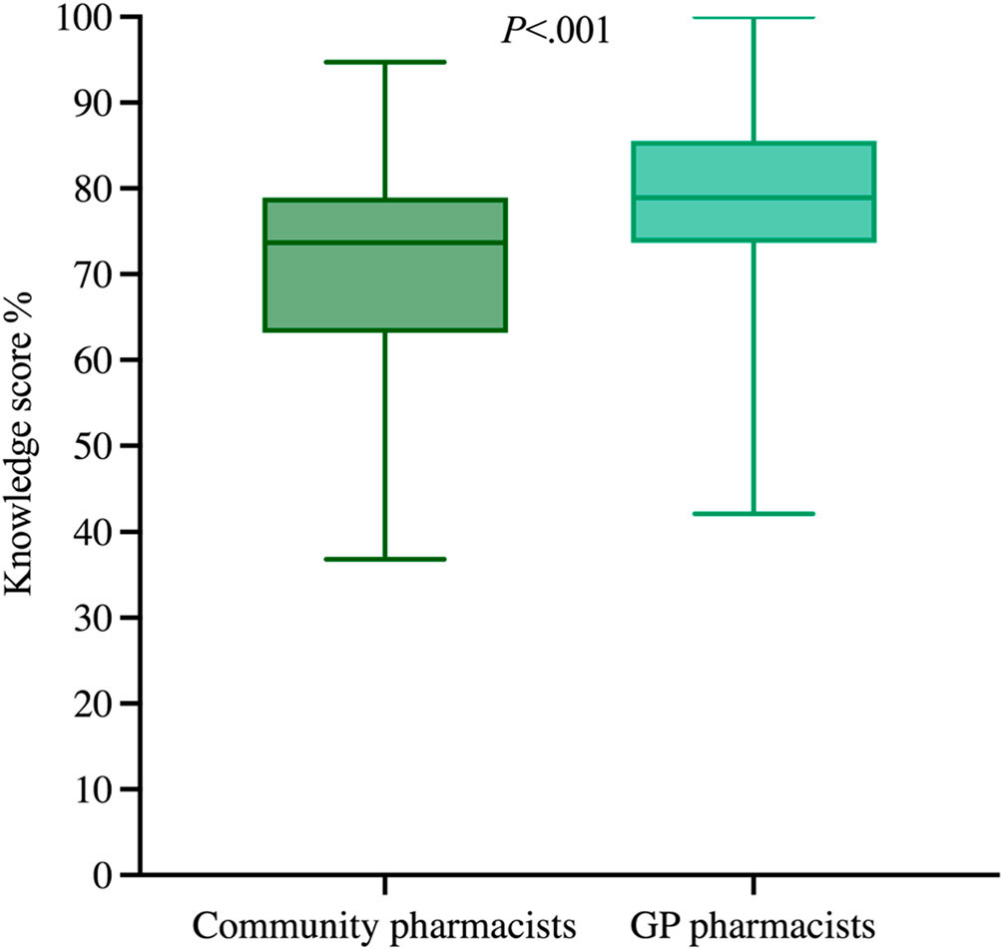
Knowledge score (%) for community (*n* = 136) and GP pharmacists (*n* = 150). GP pharmacists: General practice-based pharmacists.

The difference in the knowledge score between community pharmacists and GP pharmacists was also statistically significant. Community pharmacists had a lower knowledge score compared to GP pharmacists (mean rank 116.28 vs. 168.18; U = 6497.5, Z = −5.360, *P *< .001, *n* = 286) respectively (Figure 3).

**Figure 4.**
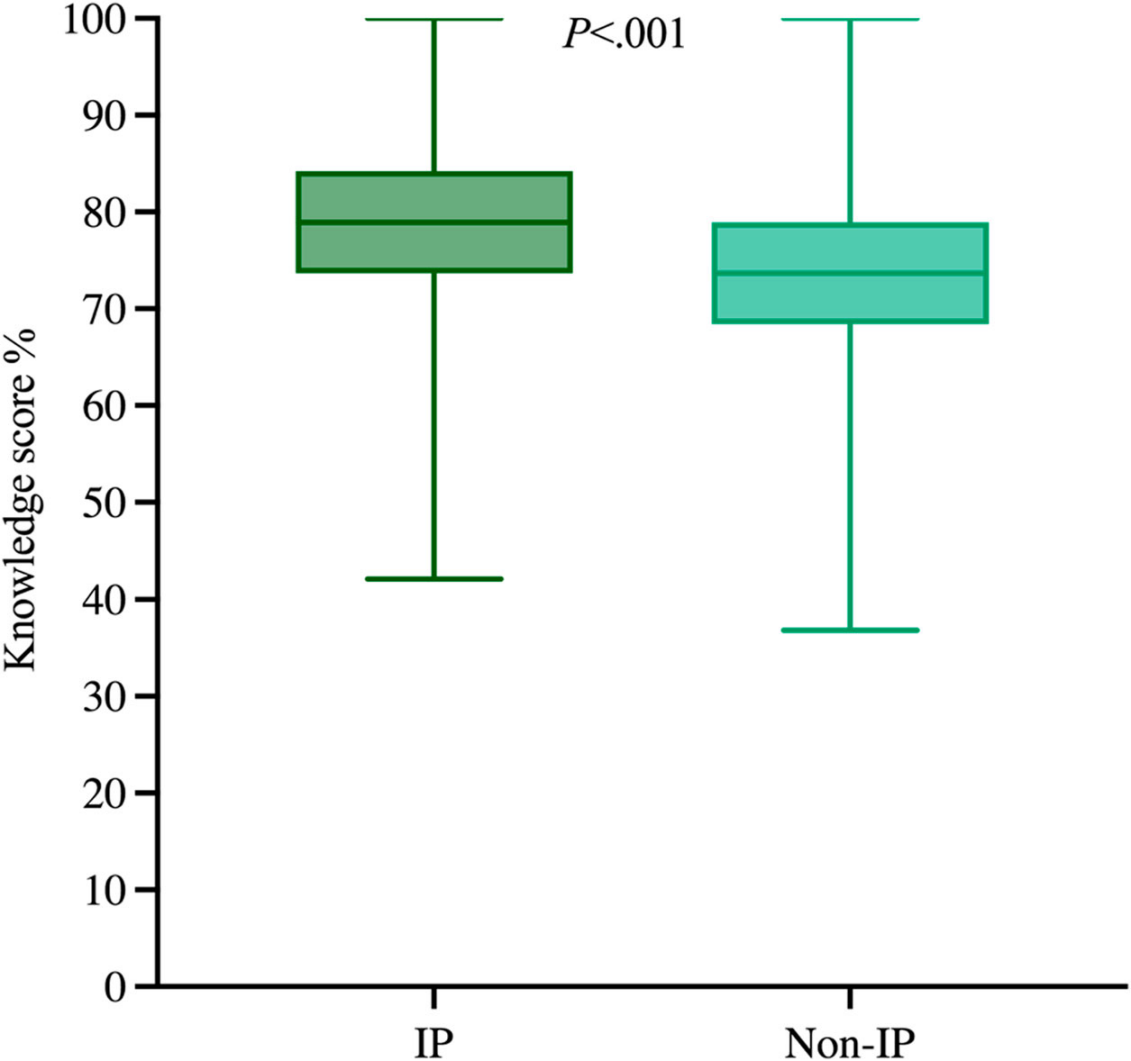
Knowledge score (%) of independent prescribers (*n* = 152) and non-independent prescribers (*n* = 192). IP: Independent prescribers; Non-IP: Non-independent prescribers. Non-IP group includes non-prescriber and supplementary prescriber.

Of the pharmacists surveyed, there was a statistically significant difference in overall knowledge scores for pharmacists who were independent prescribers when compared to those that did not have an independent prescribing qualification. Independent prescribers had a higher knowledge score compared to non-independent prescribers (mean rank 203.02 vs.153.38; *P *< .001, *n* = 349) respectively (Figure 4).

**Figure 5.**
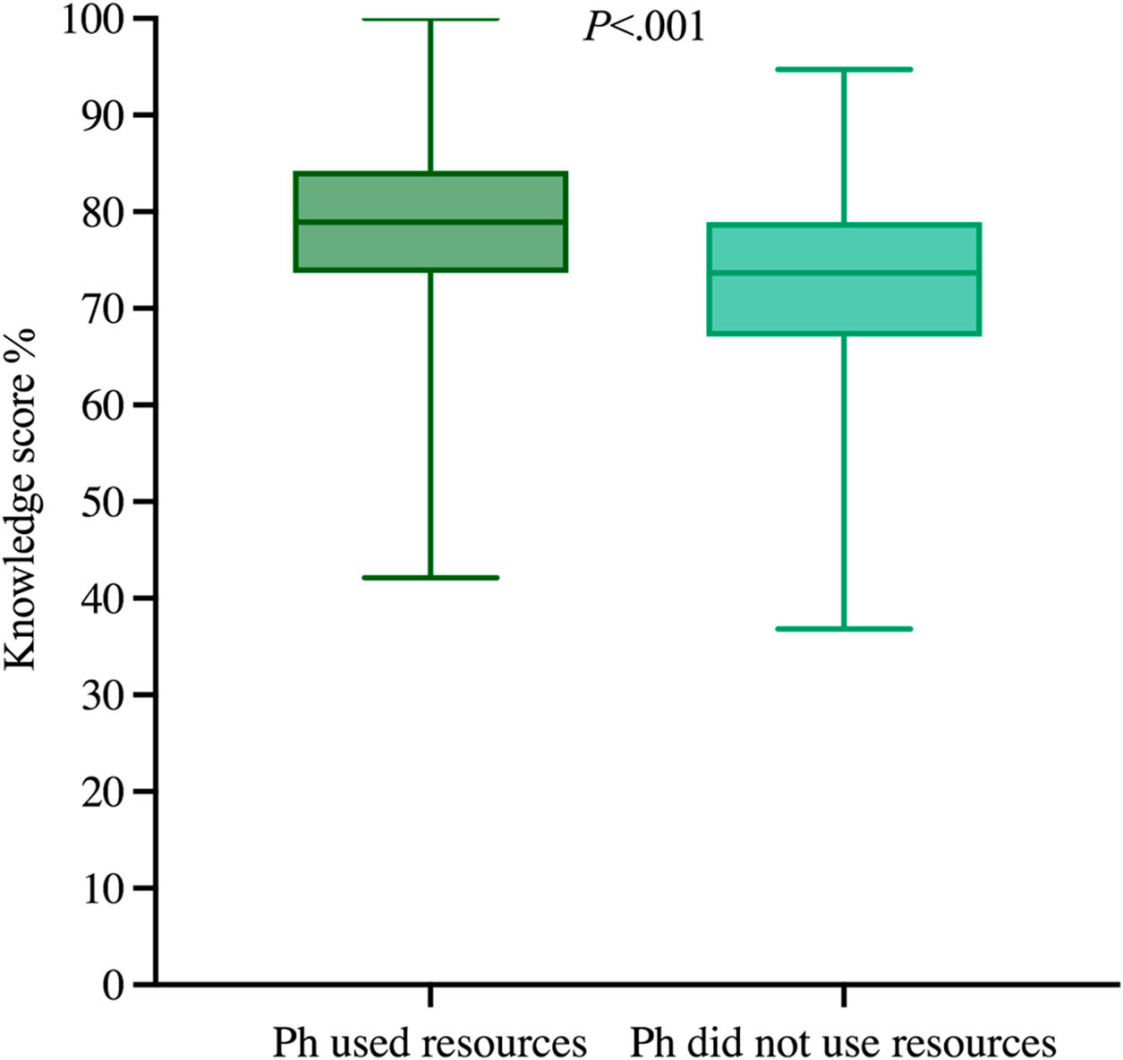
Knowledge score (%) for pharmacists who used information resources during consultations (*n* = 219) and those who did not (*n* = 130). Ph: Pharmacists.

Furthermore, the difference in the knowledge scores for those who used resources to support the consultations on anticoagulation, and those who did not, was statistically significant: pharmacists who used resources had a higher knowledge score when compared to those who did not (mean rank 195.93 vs. 139.74; U = 9651.5, Z = −5.087, *P *< .001, *n* = 349) respectively (Figure 5).

## Knowledge gaps on AF and its anticoagulation management

**Figure 6.**
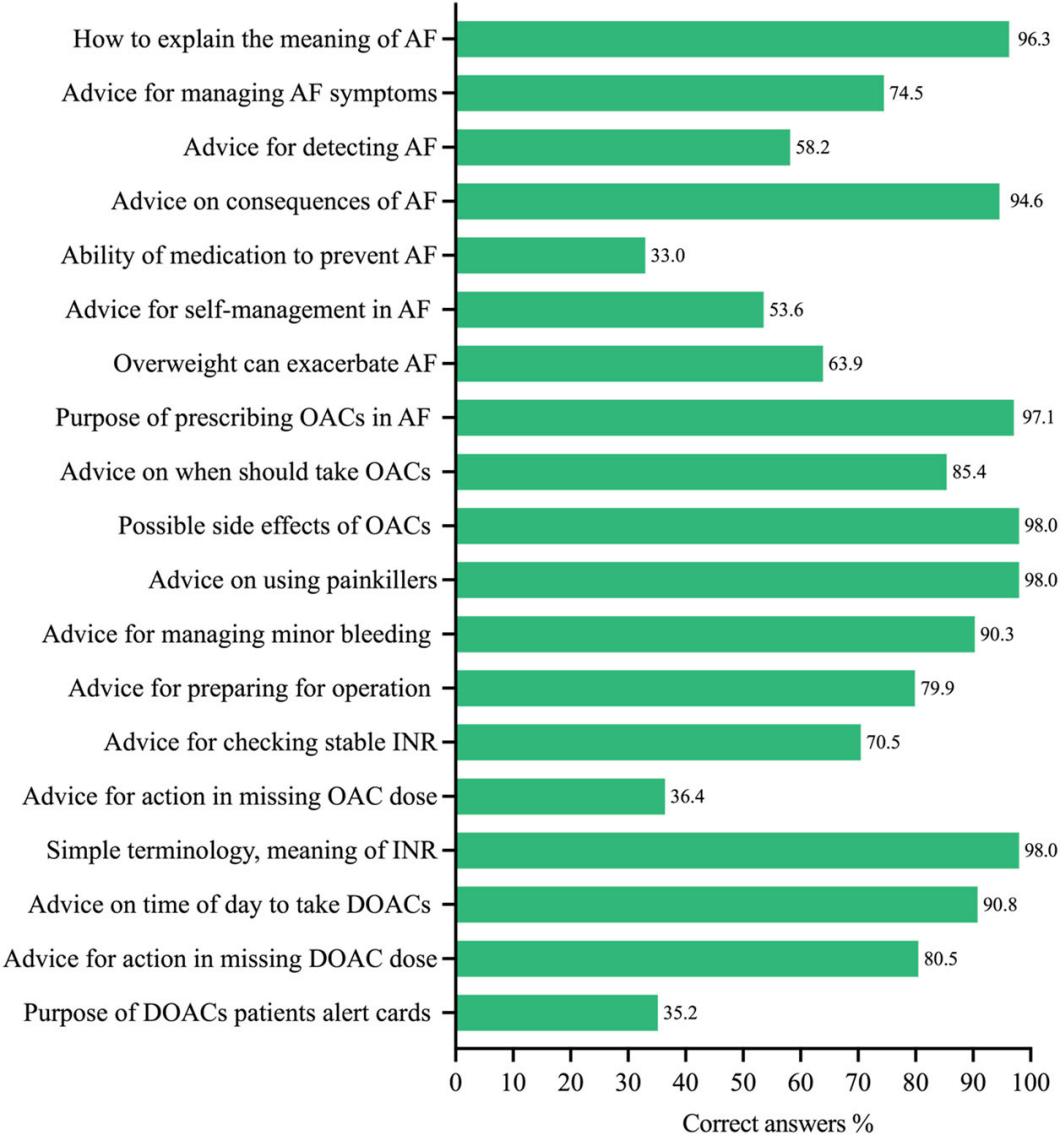
Percentages of the correct answers for the pharmacists’ knowledge questionnaire. Number of respondents for all questions (*n* = 349). DOACs: Direct oral anticoagulants; INR: International Normalised Ratio; OACs: Oral anticoagulants; VKAs: Vitamin K antagonists.

The 19 knowledge-based questions revealed several significant gaps in pharmacists’ knowledge about AF and its anticoagulation management (Figure 6). The areas that displayed these gaps included giving advice to patients on the self-management of AF. Just over half of the pharmacists (53.6%) knew that AF symptoms could be managed by the patient; and a similar proportion (58.2%) were aware that patients with AF can detect AF through a pulse check. While 63.9% were aware that being overweight can contribute to AF, only 33% of the pharmacists were aware that patients taking OACs for AF would continue to experience AF.

Generally, knowledge of how to counsel patients taking OACs was good (e.g. time of day to take the medicines, side effects, drug interactions/contraindications). The knowledge score for different types of OACs was relatively similar (mean VKA 68.30 ± 30.7% versus DOACs 68.83 ± 29.6%). However, many pharmacists surveyed had knowledge gaps around the frequency of INR monitoring for VKAs, with 70.5% aware of the right answer. Further gaps were identified around the implication of the risk of missing a dose of VKAs, and the use of DOACs patients’ alert cards with 36.4% and 35.2% of the pharmacists unable to answer these questions, respectively. Data indicated that there were knowledge deficits on the counselling items among surveyed pharmacists, which were evidenced by the low correct response rates.

## Resources to support consultations on anticoagulation

A total of 349 pharmacists completed the questions on the resources used for consultations on anticoagulation management. Almost two-thirds of the pharmacists (62.8%) reported that they used sources of information to support their consultations for patients with AF (Table 2).

**Table 2.** Pharmacist’s preference for resources to support AF consultations on anticoagulation management.

Resources	Participants % (*n*)
Did not used any resources	37.2 (130)
**Mix resources**	
British National Formulary (BNF)	50.7 (177)
National Institute for Health and Care Excellence (NICE)	52.4 (183)
**Online resources**	
Recognised healthcare website	14.0 (49)
European Society of Cardiology (ESC) guidelines	2.6 (9)
Local guidance	6.3 (22)
**Other resources**	
Manufacturer information	4.6 (16)

The most frequently mentioned resources were National Institute for Health and Care Excellence (NICE) guidance and the British National Formulary (BNF); half of the surveyed pharmacists reported that they used the NICE (52.4%) and BNF (50.7%). Online resources were accessed by 22.9% of pharmacists, of whom half specified recognised healthcare websites such as the NHS website, NHS anticoagulants, and Keele anticoagulation tool; whilst others utilised the European Society of Cardiology (ESC) guidelines and local guidelines. Only 4.6% of pharmacists used the manufacturer’s summary of product characteristics.

## Discussion

## Principal findings

Responses from 349 primary care pharmacists were received. Participants’ length of experience and professional sector varied. The level of knowledge of the pharmacists surveyed widely varied. Pharmacists’ knowledge of the management of AF and anticoagulation was good. The knowledge of different types of OACs (VKAs versus DOACs) was similar. However, knowledge gaps in certain areas existed, such as advice on actions to take when on OAC dose is missed, and also advice on the purpose of the DOAC alert card. Most of the surveyed pharmacists, not all, used resources to support the consultations on anticoagulation, and these pharmacists had better general knowledge about AF management. The most frequently mentioned of these resources were National Institute for Health and Care Excellence (NICE) guidance and the British National Formulary (BNF).

## Comparison with previous published literature

Previous studies evaluating the impact of pharmacist management of anticoagulation have reported promising findings with regard to maintaining INR values within the therapeutic range (Young et al., 2011), as well as decreasing the incidence of ischaemic and haemorrhagic stroke events in warfarin management (Bungard et al., 2009). Further, studies reported pharmacists’ management of DOACs had a positive impact on increasing appropriate dosing (Ashjian et al., 2017) and medication adherence (Shore et al., 2015). However, a recent multinational study researching pharmacist management of anticoagulation reported that pharmacists felt more confident in supporting patients on VKAs compared to DOACs (Papastergiou et al., 2017). This may be a reflection of their considerable experience with VKAs, which have been the mainstay of anticoagulation therapy for a long period.

Literature suggests that confidence is defined as the ability to take knowledge and skill and move it into action (Center & Adams, 2013), and is valued, in its role in developing competence, as well as being the ultimate measure for demonstrating competence (Cohen et al., 2013). However, confidence does not always guarantee competence (Olsson, 2014). Typically, competence refers to the possession of the knowledge, skills and attitudes that enable the provision of quality care, appropriate behaviours, and sound clinical judgments. This suggests that knowledge, confidence, and competence are all interrelated and complementary as knowledge, alongside confidence, is crucial in producing competence. The present study assessed the actual pharmacists’ knowledge on the management of AF and anticoagulation and reported a variation level of knowledge among surveyed pharmacists, which was affected by many aspects, such as professional sector experiences, independent prescribing qualifications, and whether pharmacists used resources. This finding may be explained by the nature of the work in daily practice; for example, the interprofessional environment and exposure to a variety of tasks for GP pharmacists may explain their higher knowledge score. In contrast, pharmacists in the community pharmacies tend largely to be engaged in dispensing practices.

The present study reported that knowledge on different types of OACs (i.e. VKAs versus DOACs) was relatively similar; this aligns with the finding of the educational intervention study (Al-Arkee et al., 2021), and which showed very similar values for pharmacists’ knowledge scores. This finding is understandable as VKAs have been available for a long time, which suggests that pharmacists’ knowledge of its use would be high; yet the update of national and international guidelines for the anticoagulation management of AF in 2016, which now recommend that new patients start on a DOAC (European Society of Cardiology [ESC], 2020; NICE, 2021), has led to higher prescription of DOACs within the last five years (van den Heuvel et al., 2018). This exposure of pharmacists to DOACs management may be reflected in their knowledge of DOACs use.

The present study shows that knowledge gaps exist in certain areas. This may explain the findings of the previous quantitative studies which assessed the confidence among pharmacists on the anticoagulation management, and reported the lowest confidence in advising on the management of bleeding and missed dose management (Ghadrdan et al., 2022), INR monitoring and making dose recommendation (Papastergiou et al., 2017), and patients’ alert cards (Hamedi et al., 2017); which are similar areas to the knowledge gaps in the present study. This finding may suggest a relation between pharmacists’ confidence and pharmacists’ actual knowledge.

The present study found that most of the surveyed pharmacists used resources to support their consultations, but not all. The study also found that a variety of information resources were used to support anticoagulation management in patients with AF; with the NICE guidelines and the BNF as the most common resources used. This finding is in line with a previous quantitative study which reported the BNF as a predominant resource used by community pharmacists to support their role in anticoagulation management in England (Hamedi et al., 2017). These resources contain clinical recommendations, and focus on the effectiveness and safety of medicines, but lack information related to practical advice for pharmacists (e.g. process and factors influencing on medication adherence) which may be required to support patients receiving anticoagulation therapy. Furthermore, the present study reported a diversity of online resources accessed. This may be an issue as using many sources may potentially lead to variation in the standard of information provided. The standard of information is not related to the quality of information but rather to consistency.

## Implications for research and practice

Pharmacists, as experts of medicines and healthcare, especially those based in primary care, may be well placed to support anticoagulation management in patients with AF. However, data presented in this study suggests that surveyed pharmacists have gaps in knowledge in this therapeutic area. Thus, imparting further education to pharmacists on the management of AF and anticoagulation may help in improving their knowledge, and ultimately their confidence. Future research should focus on assessing pharmacists’ basic knowledge of the management of AF and anticoagulation, imparting relevant education, and assessing the impact of such activity.

## Strengths and limitations

To the author’s best knowledge, this is the only quantitative study that has assessed the actual level of UK primary care pharmacists’ knowledge of AF and associated anticoagulation management, rather than assessing their level of confidence. The detailed demographic data enabled a more accurate comparison between pharmacists’ groups. The participation of both community and GP pharmacists enabled the level of knowledge to be assessed in both groups, thus providing an overall picture of the pharmacists’ knowledge of AF and anticoagulation management in primary care. However, the sampling method was a limitation. This was due to the lack of stratified random sampling to guarantee a sample population to best represent the entire population with homogenous groups (i.e. community pharmacists and GP pharmacists). Thus, the findings may not be nationally representative or provide a holistic picture.

Sample size was a strength compared to existing literature, as the sample size exceeded the estimation. However, there was a limitation, after conducting the research, a sample size calculation was performed which was based on the actual number of UK pharmacists. In 2019, the number of pharmacists working in the UK was approximately 62,800 (Mikulic, 2021). Assuming that most of those were working in primary care and could deliver clinical consultations on anticoagulation, using a confidence interval of 95% and accepting a 5% error, approximately 382 responses were anticipated.

The tool used was another limitation, whereby this study used a survey informed by existing literature, which included a section with the practical advice that pharmacists may need in daily practice to support anticoagulation management in patients with AF. However, the development process of the survey did not include testing construct validity and internal reliability (e.g. psychometric properties of the survey) with targeted population (i.e. primary care pharmacists). Thus, conclusions around pharmacists’ knowledge cannot be made with complete confidence.

## Conclusion

Primary care pharmacists are knowledgeable with regard to the preventative measure for the potential adverse events of the AF condition; yet some gaps exist which may require addressing. Although pharmacists use a variety of information resources to support anticoagulation management in patients with AF within daily practice, it is the traditional resources that remain dominant.

## Supplementary Material

Supplemental Material

## Data Availability

All data generated or analysed during this study are included in this published article [and its Additional files]. Supplementary data related to this study can be found at Additional file 1.

## Abbreviations

**Abbreviations:** AF, Atrial Fibrillation; Apps, Mobile Applications; BNF, British National Formulary; CVD, Cardiovascular Diseases; DOACs, Direct Oral Anticoagulants; GPs, General Practices (GPs); HCPs, Healthcare Professionals; NHS, National Health Service; NICE, National Institute for Health and Care Excellence; OACs, Oral Anticoagulants; PMR, Patient Medical Records; UOB, University of Birmingham; VKAs, Vitamin K antagonists

## References

[CIT0001] Al-Arkee, S., Mason, J., Fabritz, L., Chua, W., Lane, D., & Jalal, Z. (2021). Pharmacist management of atrial fibrillation: A pilot educational intervention study. *European Heart Journal*, *42*(Supplement_1), ehab724.0544. 10.1093/eurheartj/ehab724.0544

[CIT0002] Ashjian, E., Kurtz, B., Renner, E., Yeshe, R., & Barnes, G. D. (2017). Evaluation of a pharmacist-led outpatient direct oral anticoagulant service. *American Journal of Health-System Pharmacy*, *74*(7), 483–489. 10.2146/ajhp151026 28336758

[CIT0003] Bungard, T. J., Gardner, L., Archer, S. L., Hamilton, P., Ritchie, B., Tymchak, W., & Tsuyuki, R. T. (2009). Evaluation of a pharmacist-managed anticoagulation clinic: Improving patient care. *Open Medicine*, *3*(1), e16–e21. 19946388 PMC2765765

[CIT0004] Center, D. L., & Adams, T. M. (2013). Developing confidence decreases guessing and increases competency. *The Journal of Continuing Education in Nursing*, *44*(9), 389–390. 10.3928/00220124-20130823-35 24015795

[CIT0005] Cohen, E. R., Barsuk, J. H., McGaghie, W. C., & Wayne, D. B. (2013). Raising the bar: Reassessing standards for procedural competence. *Teaching and Learning in Medicine*, *25*(1), 6–9. 10.1080/10401334.2012.741540 23330888

[CIT0006] Collins, S., Barber, A., & Sahm, L. J. (2014). Pharmacist’s counselling improves patient knowledge regarding warfarin, irrespective of health literacy level. *Pharmacy*, *2*(1), 114–123. 10.3390/pharmacy2010114

[CIT0007] Connolly, S. J., Ezekowitz, M. D., Yusuf, S., Eikelboom, J., Oldgren, J., Parekh, A., Pogue, J., Reilly, P. A., Themeles, E., Varrone, J., Wang, S., Alings, M., Xavier, D., Zhu, J., Diaz, R., Lewis, B. S., Darius, H., Diener, H.-C., Joyner, C. D., & Wallentin, L. (2009). Dabigatran versus warfarin in patients with atrial fibrillation. *New England Journal of Medicine*, *361*(12), 1139–1151. 10.1056/NEJMoa0905561 19717844

[CIT0008] Desteghe, L., Engelhard, L., Raymaekers, Z., Kluts, K., Vijgen, J., Dilling-Boer, D., Koopman, P., Schurmans, J., Dendale, P., & Heidbuchel, H. (2016). Knowledge gaps in patients with atrial fibrillation revealed by a new validated knowledge questionnaire. *International Journal of Cardiology*, *223*, 906–914. 10.1016/j.ijcard.2016.08.303 27589038

[CIT0009] European Society of Cardiology [ESC]. (2020). *2020 Guidelines for management of atrial fibrillation*. ESC Clinical Practice Guidelines. https://www.escardio.org/Guidelines/Clinical-Practice-Guidelines/Atrial-Fibrillation-Management

[CIT0010] Ghadrdan, E., Mohammadi, M., Namazi, S., Daie, M., & Ebrahimpour, S. (2022). Assessment of pharmacists’ confidence when consulting patients on anticoagulants: A cross-sectional study in Iran. *Journal of Pharmaceutical Care*, *10*(1), 28–33.

[CIT0011] Giugliano, R. P., Ruff, C. T., Braunwald, E., Murphy, S. A., Wiviott, S. D., Halperin, J. L., Waldo, A. L., Ezekowitz, M. D., Weitz, J. I., Špinar, J., Ruzyllo, W., Ruda, M., Koretsune, Y., Betcher, J., Shi, M., Grip, L. T., Patel, S. P., Patel, I., Hanyok, J. J., … Antman, E. M. (2013). Edoxaban versus warfarin in patients with atrial fibrillation. *New England Journal of Medicine*, *369*(22), 2093–2104. 10.1056/NEJMoa1310907 24251359

[CIT0012] Granger, C. B., Alexander, J. H., McMurray, J. J., Lopes, R. D., Hylek, E. M., Hanna, M., Al-Khalidi, H. R., Ansell, J., Atar, D., Avezum, A., Bahit, M. C., Diaz, R., Easton, J. D., Ezekowitz, J. A., Flaker, G., Garcia, D., Geraldes, M., Gersh, B. J., Golitsyn, S., … Wallentin, L. (2011). Apixaban versus warfarin in patients with atrial fibrillation. *New England Journal of Medicine*, *365*(11), 981–992. 10.1056/NEJMoa1107039 21870978

[CIT0013] GraphPad. (2022). www.graphpad.com.

[CIT0014] Gupta, V., Kogut, S. J., & Thompson, S. (2015). Evaluation of differences in percentage of international normalized ratios in range between pharmacist-led and physician-led anticoagulation management services. *Journal of Pharmacy Practice*, *28*(3), 249–255. 10.1177/0897190013516368 24381239

[CIT0015] Hamedi, N., da Costa, F. A., Horne, R., Levitan, M., Begley, A., & Antoniou, S. (2017). How prepared are pharmacists to support atrial fibrillation patients in adhering to newly prescribed oral anticoagulants? *International Journal of Clinical Pharmacy*, *39*(6), 1273–1281. 10.1007/s11096-017-0529-0 28875370

[CIT0016] Hart, R. G., Pearce, L. A., & Aguilar, M. I. (2007). Meta-analysis: Antithrombotic therapy to prevent stroke in patients who have nonvalvular atrial fibrillation. *Annals of Internal Medicine*, *146*(12), 857–867. 10.7326/0003-4819-146-12-200706190-00007 17577005

[CIT0017] Information Commissioner’s Office. (2018). *Guide to the General Data Protection Regulation (GDPR)*. https://ico.org.uk/media/for-organisations/guide-to-the-general-data-protection-regulation-gdpr-1-0.pdf.

[CIT0018] Kirchhof, P., Benussi, S., Kotecha, D., Ahlsson, A., Atar, D., Casadei, B., Castella, M., Diener, H.-C., Heidbuchel, H., Hendriks, J., Hindricks, G., Manolis, A. S., Oldgren, J., Popescu, B. A., Schotten, U., Van Putte, B., Vardas, P., Agewall, S., Camm, J., … Vardas, P. (2016). 2016 ESC guidelines for the management of atrial fibrillation developed in collaboration with EACTS. *European Heart Journal*, *37*(38), 2893–2962. 10.1093/eurheartj/ehw210 27567408

[CIT0019] Mikulic, M. (2021). *Annual number of pharmacists in the United Kingdom (UK) from 2010 to 2021**. https://www.statista.com/statistics/318874/numbers-of-pharmacists-in-the-uk/

[CIT0020] National Institute for Health and Care Excellence [NICE]. (2021). *Atrial fibrillation: Diagnosis and management [NG196]*. https://www.nice.org.uk/guidance/ng196 34165935

[CIT0021] Olsson, H. (2014). Measuring overconfidence: Methodological problems and statistical artifacts. *Journal of Business Research*, *67*(8), 1766–1770. 10.1016/j.jbusres.2014.03.002

[CIT0022] Papastergiou, J., Kheir, N., Ladova, K., Rydant, S., De Rango, F., Antoniou, S., Viola, R., Murillo, M. D., Steurbaut, S., & da Costa, F. A. (2017). Pharmacists’ confidence when providing pharmaceutical care on anticoagulants, a multinational survey. *International Journal of Clinical Pharmacy*, *39*(6), 1282–1290. 10.1007/s11096-017-0551-2 29139019 PMC5694509

[CIT0023] Patel, M. R., Mahaffey, K. W., Garg, J., Pan, G., Singer, D. E., Hacke, W., Breithardt, G., Halperin, J. L., Hankey, G. J., Piccini, J. P., Becker, R. C., Nessel, C. C., Paolini, J. F., Berkowitz, S. D., Fox, K. A. A., & Califf, R. M. (2011). Rivaroxaban versus warfarin in nonvalvular atrial fibrillation. *New England Journal of Medicine*, *365*(10), 883–891. 10.1056/NEJMoa1009638 21830957

[CIT0024] Peat, J. K., Mellis, C., Williams, K., & Xuan, W. (2020). *Health science research: A handbook of quantitative methods*. Routledge.

[CIT0025] Rudd, K. M., & Dier, J. G. (2010). Comparison of two different models of anticoagulation management services with usual medical care. *Pharmacotherapy: The Journal of Human Pharmacology and Drug Therapy*, *30*(4), 330–338. 10.1592/phco.30.4.330 20334453

[CIT0026] Shore, S., Ho, P. M., Lambert-Kerzner, A., Glorioso, T. J., Carey, E. P., Cunningham, F., Longo, L., Jackevicius, C., Rose, A., & Turakhia, M. P. (2015). Site-level variation in and practices associated with dabigatran adherence. *JAMA*, *313*(14), 1443–1450. 10.1001/jama.2015.2761 25871670

[CIT0027] Stewart, S., Murphy, N. F., Walker, A., McGuire, A., & McMurray, J. J. (2004). Cost of an emerging epidemic: An economic analysis of atrial fibrillation in the UK. *Heart*, *90*(3), 286–292. 10.1136/hrt.2002.008748 14966048 PMC1768125

[CIT0028] Tan, S. L., Yong, Z. Y., Liew, J. E. S., Zainal, H., & Siddiqui, S. (2021). How confident are pharmacists in providing pharmaceutical care on anticoagulants? A cross-sectional, self-administered questionnaire study in Borneo, Malaysia. *Journal of Pharmaceutical Policy and Practice*, *14*(1), 97. 10.1186/s40545-021-00377-w 34753518 PMC8576986

[CIT0029] van den Heuvel, J. M., Hövels, A. M., Büller, H. R., Mantel-Teeuwisse, A. K., de Boer, A., & Maitland-van der Zee, A. H. (2018). NOACs replace VKA as preferred oral anticoagulant among new patients: A drug utilization study in 560 pharmacies in The Netherlands. *Thrombosis Journal*, *16*(1), 7. 10.1186/s12959-017-0156-y 29692686 PMC5905161

[CIT0030] Wolf, P. A., Abbott, R. D., & Kannel, W. B. (1987). Atrial fibrillation: A major contributor to stroke in the elderly. *Archives of Internal Medicine*, *147*(9), 1561–1564. 10.1001/archinte.1987.00370090041008 3632164

[CIT0031] Young, S., Bishop, L., Twells, L., Dillon, C., Hawboldt, J., & O'Shea, P. (2011). Comparison of pharmacist managed anticoagulation with usual medical care in a family medicine clinic. *BMC Family Practice*, *12*(1), 88. 10.1186/1471-2296-12-88 21849052 PMC3176160

