# Supplementary material for: Pharmacist management of atrial fibrillation in UK primary care: a cross-sectional study
Source: J Pharm Policy Pract. 2024 Mar 20;17(1):2321592. doi: 10.1080/20523211.2024.2321592 (PMC10956929; doi:10.1080/20523211.2024.2321592)
Supplement: Supplemental Material [file JPPP_A_2321592_SM8979.docx]

# Additional file 1. Primary care pharmacists’ survey.

*Section one: Demographic data*

# Please tell us a bit about you.

- 1. **What is your gender?**
     - Male
     - Female
     - Other
     - Prefer not to say

# What year did you first register as a pharmacist in the United Kingdom (UK)?

- 1. **Have you practiced as a pharmacist in any other countries outside the UK?**
     - No
     - Yes, please specify:

1. The country
2. How long you practiced outside the UK?

……. years

# How long have you been practicing as a pharmacist in the UK?

* Please specify

……. years

# Have you taken any career break where you have not practiced as a pharmacist?

- - - No
    - Yes, if you selected yes. please specify:

a. How long?

……. years

- 1. **What is the pharmacy sector that are you currently working in** (e.g., Academia, Community, General practice, Hospital, Industry, Other)**?**

# Have you spent any time working in other sectors of pharmacy?

- - - No
    - Yes, please specify:

1. The sector
2. How long have you practiced in this sector (e.g., Academia, industry, hospital, etc.)?

……. years

# Have you undertaken any postgraduate qualifications?

- - - None
    - Diploma *please specify (e.g., Community practice)
    - Master *please specify (e.g., Clinical pharmacy)
    - PhD *please specify (e.g., Pharmacy practice)
    - Other, please specify

# Are you a prescriber?

- - - No
    - Yes, I am an independent prescriber
    - Yes, I am a supplementary prescriber If yes, please specify your specialist area

# Have you undertaken any postgraduate courses in order to further develop your consultation skills?

- - - None
    - Yes, please specify:

1. What were they?
2. What qualifications did they lead to (if any)?
3. Did they cover atrial fibrillation, or it is management?

# Do you use any resources when you counselling a patient with atrial fibrillation?

***** Please tick all that apply

- No
- British National Formulary (BNF)
- The National Institute for Heath and Care Excellence (NICE)
- Other, please list the resources used

*Section Two: Pharmacists’ knowledge*

# Questions about atrial fibrillation in general

- 1. **How do you explain the meaning of atrial fibrillation to a patient?**
     - A condition where the heart beats irregularly and often faster than normal
     - A heart muscle disease through which the heart is unable to pump enough blood through the body
     - A blood disorder causing the development of clots in the heart
     - I do not know

# Is atrial fibrillation always accompanied by symptoms?

- - - Yes
    - Yes, but only in patients with a certain degree of heart failure
    - No
    - I do not know

# What would you advise a patient to do to detect atrial fibrillation?

- - - Nothing, atrial fibrillation is very difficult to detect and only a doctor can recognise the condition
    - Take their pulse regularly.
    - Get a urine test done at a local pharmacy
    - I do not know
    - Other, please specify

# What are the consequences of atrial fibrillation?

- - - It has no harmful effects
    - It can cause blood clots which can lead to stroke (cerebral infarction)
    - It can lead to increased blood pressure, causing the body to retain fluid
    - I do not know

# Medication can:

- - - Permanently prevent atrial fibrillation, so that the arrhythmia will never come back
    - Largely prevent the atrial fibrillation: the arrhythmia will only occur sporadically
    - Not prevent atrial fibrillation permanently, as arrhythmia will increasingly occur with ageing, even when taking medication
    - I do not know

# Would you advise a patient to go to general practitioner or emergency room each time when he/she feels atrial fibrillation?

- - - Yes
    - No
    - Only at night or on the weekend
    - I do not know
    - Other, please specify

# The patient being overweight:

- - - Exacerbates atrial fibrillation
    - Has no effect on atrial fibrillation
    - Protects against atrial fibrillation
    - I do not know

# Why are blood thinners (oral anticoagulants) often prescribed for the patient with atrial fibrillation?

- - - In order to prevent the body from retaining fluid
    - In order to prevent the development of blood clots in the heart, which can lead to stroke
    - In order to allow blood to flow more easily throughout the body and hence lower blood pressure
    - I do not know

# Questions about blood thinners (oral anticoagulants) for patients with atrial fibrillation

- 1. **When should the patient take blood thinners?**
     - Patient should only take blood thinners if they have had a stroke
     - Only when a patient feels atrial fibrillation, for one week
     - A patient should always take blood thinners, even if they do not feel atrial fibrillation
     - I do not know

# Possible side effects of blood thinners?

- - - Headaches and dizziness
    - A too low or too high blood pressure
    - The occurrence of bleedings and longer bleeding time in case of injuries
    - I do not know
    - Other, please specify

# Which painkillers may you advise the patient to take?

- - - Any painkiller
    - Medication based on paracetamol: e.g., Panadol
    - One of the following anti-inflammatory agents: e.g., Aspirin, Ibuprofen
    - I do not know

# What do you advise a patient to do if he/she regularly has minor nose bleeds (that spontaneously cease)?

- - - Patient should stop talking blood thinners
    - Patient should contact the general practitioner or specialist, while continuing to take blood thinners
    - Patient should go to the emergency service
    - I do not know
    - Other, please specify

# What if the patient needs an operation?

- - - Patient can go on taking blood thinners
    - Patient should discuss the options with the doctor
    - Patient should stop taking blood thinners one week in advance
    - I do not know

# Questions about vitamin K antagonists (VKAs)

- 1. **How often should patients with a stable INR have their blood thinning checked?**
     - At least once a month
     - At least once every 12 weeks
     - Twice a year
     - Once a year
     - I do not know
     - Other, please specify

# What should a patient do if they have forgotten to take their blood thinner?

- - - Patient should still take the forgotten pill (immediately or at the next dose)
    - Patient should skip that dose and wait until the next dose
    - Patient should take a regular aspirin instead
    - I do not know

# Using simple terminology, what does INR (International Normalised Ratio) mean?

- - - It is a measure to check how well your kidneys work
    - It is a measure to check whether you have anaemia
    - It is a measure to check how thick or how thin your blood is
    - I do not know
    - Other, please specify

# Questions about new blood thinners (non-vitamin K antagonist oral anticoagulants: NOACs) (e.g., Apixaban, Dabigatran, Edoxaban, and Rivaroxaban)

- 1. **Is it important that a patient takes their blood thinner at the same time every day?**
     - Yes
     - No
     - No, as long as it is between meals
     - I do not know

# What should a patient do if they have forgotten to take their blood thinner?

- - - Patient should still take that dose, unless the time till their next dose is less than the time after their missed dose
    - Patient should skip a dose and wait until the next dose
    - Patient should take two pills at the next dose
    - I do not know

# The blood thinner comes with a card. What should the patient do with the card?

- - - Indicate when he or she takes the blood thinner it
    - Show it to the general practitioner and specialist
    - Give it to the pharmacist so that the pharmacist can give the right medication
    - I do not know
